# Methyl Jasmonate Alleviates Cadmium-Induced Photosynthetic Damages through Increased S-Assimilation and Glutathione Production in Mustard

**DOI:** 10.3389/fpls.2016.01933

**Published:** 2016-12-22

**Authors:** Tasir S. Per, Nafees A. Khan, Asim Masood, Mehar Fatma

**Affiliations:** Plant Physiology and Biochemistry Laboratory, Department of Botany, Aligarh Muslim UniversityAligarh, India

**Keywords:** cadmium toxicity, glutathione, methyl jasmonate, oxidative stress, photosynthesis

## Abstract

The effect of methyl jasmonate (MeJA) in mitigation of 50 μM cadmium (Cd) toxicity on structure and function of photosynthetic apparatus in presence or absence of 1.0 mM ${\mathrm{SO}}_{4}^{2–}$ was investigated in mustard (*Brassica juncea* L. cv. Ro Agro 4001) at 30 days after sowing. Plants exhibited increased oxidative stress, impaired photosynthetic function when grown with Cd, but MeJA in presence of sulfur (S) more prominently ameliorated Cd effects through increased S-assimilation and production of reduced glutathione (GSH) and promoted photosynthetic functions. The transmission electron microscopy showed that MeJA protected chloroplast structure against Cd-toxicity. The use of GSH biosynthetic inhibitor, buthionine sulfoximine (BSO) substantiated the findings that ameliorating effect of MeJA was through GSH production. MeJA could not alleviate Cd effects when BSO was used due to unavailability of GSH even with the input of S. The study shows that MeJA regulates S-assimilation and GSH production for protection of structure and function of photosynthetic apparatus in mustard plants under Cd stress.

## Introduction

Cadmium (Cd) is a non-essential toxic heavy metal and an important agricultural pollutant due to its adverse effects on metabolic processes, such as membrane damage, alteration in electron transport, activation or inhibition of enzymes activity, and DNA damage (Gallego et al., 2012; Asgher et al., 2015). Cd even at low concentration directly or indirectly targets photosynthesis and strongly inhibits growth and development (Andresen and Küpper, 2013). The indirect consequence of Cd toxicity is overproduction of reactive oxygen species (ROS); super oxide radical ($O_{2}^{∙-}$), hydroxyl radical (^∙^OH), and hydrogen peroxide (H_2_O_2_) (Andresen and Küpper, 2013). The higher generation of ROS accelerates lipid peroxidation by altering the composition of membrane lipids due to the formation of malondialdehyde (MDA) (Gill and Tuteja, 2010; Tian et al., 2012). Exposure of plants to Cd is at high risk due to the increasing industrial emissions, application of phosphatic fertilizers and sewage sludge to farm land. Thus, minimizing Cd concentration in crop harvests is crucial in order to curtail the hazardous effects of Cd on crop productivity and human health. Plants exploit several strategies to rescue from the oxidative stress caused by ROS. For this purpose, several efficient ROS-scavenging enzymes, superoxide dismutase (SOD, EC; 1.15.1.1), ascorbate peroxidase (APX, EC; 1.11.1.11) and glutathione reductase (GR, EC; 1.6.4.2), and non-enzymatic components, ascorbic acid (AsA) and reduced glutathione (GSH) are triggered to avoid the adverse effects of ROS (Gratão et al., 2008; Foyer and Noctor, 2012; Masood et al., 2012; Roychoudhury et al., 2012; Asgher et al., 2014; Singh and Shah, 2014).

Sulfur (S) is an important nutrient element and constituent of S-containing amino acids, cysteine (Cys), and methionine (Met) which take part in the synthesis of GSH in S-assimilation pathway. GSH is the major antioxidant in plant stress defense and play an important role in trace element homeostasis (Na and Salt, 2011; Capaldi et al., 2015). Uptake and assimilation of S is critical for determining crop yield and resistance to Cd stress (Gallego et al., 2012; Koprivova and Kopriva, 2014). Most agricultural soils are naturally low in fertility and can be improved by supplementation of essential mineral nutrients such as nitrogen (N), phosphorus (P), potassium (K), and S. The inadequacy of soil S can also cause the inefficient use of other nutrients, such as carbon and N leading to deficiency and decrease in protein biosynthesis, chlorophyll content and crop yield (Capaldi et al., 2015).

Plant growth hormones, auxin, gibberellins, cytokinin, abscisic acid, brassinosteroid, and jasmonic acid (JA) act as components of abiotic-stress signaling (Wani et al., 2016). JA and its methyl ester, methyl jasmonate (MeJA) are naturally occurring plant growth regulators that affect various physiological and biochemical processes in plants (Wang, 1999; Ueda and Saniewski, 2006; Norastehnia et al., 2007). Contradictory reports are available regarding the role of MeJA in protection of plants from various abiotic stresses. MeJA is generally considered to inhibit stomatal opening, photosynthetic activity and plant growth (Anjum et al., 2011; Yan et al., 2015). On the other hand, reports show that MeJA at low concentrations improves physiological responses against abiotic stress factors (Walia et al., 2007; Keramat et al., 2009). It has been found that jasmonates and MeJA counteract the toxic effects of Cd and lead (Pb) in plants (Keramat et al., 2009; Moussa and El-Gamal, 2010; Singh and Shah, 2014). Some studies have indicated that MeJA applied at 10^-7^ to 10^-5^ M exerts a stimulatory effect on photosynthetic pigments, photosystem II (PSII) activity that were impaired due to toxic metals (Maksymiec and Krupa, 2002; Kováčik et al., 2011). These relieving effects by JA or MeJA were associated with the enhancement of antioxidant capacity and the reduced content of thiobarbituric acid reactive substances (TBARS) and H_2_O_2_ (Keramat et al., 2009; Kováčik et al., 2011; Noriega et al., 2012; Singh and Shah, 2014). JA has been found to activate genes involved in the signal transduction pathway for copper (Cu) and Cd, up-regulated GSH-metabolic genes and potentially enhanced synthesis of GSH. Xiang and Oliver (1998) have reported that JA-induced tolerance was largely controlled at the transcriptional level by increasing mRNA level in *Arabidopsis thaliana*. Jasmonates also enhanced the content of GSH in *Glycine max* and *Oryza sativa* under Cd stress (Noriega et al., 2012; Singh and Shah, 2014).

Mustard (*Brassica juncea* L.), an important oilseed crop which exhibits higher tolerance toward most of the toxic metals/metalloids and majority of *Brassica* species are now known as good accumulators of toxic metals including Cd (Nouairi et al., 2006). Plants (including *B. juncea*) have different enzymatic mechanisms that jointly with other defense compounds play a crucial role in mitigating the toxic effects of heavy metals. In the present study, an effort was made to elucidate the effect of MeJA in S-mediated influence on photosynthetic responses and antioxidant system in mustard in presence or absence of Cd stress. First of all, the effects of MeJA on oxidative stress and photosynthesis were investigated in presence or absence of Cd, followed by studying the effects of MeJA with or without S in the alleviation of Cd toxicity. The involvement of GSH in MeJA-mediated Cd stress tolerance using GSH biosynthetic inhibitor, buthionine sulfoximine (BSO), was also studied. Our results show that MeJA enhances tolerance to oxidative stress by increasing S-assimilation and GSH production, and improves photosynthetic performance in mustard.

## Materials and Methods

### Plant Material and Growth Conditions

Seeds of mustard (*B. juncea* L. cv. Ro Agro 4001) were surface sterilized with 0.01% HgCl_2_ solution, rinsed with double distilled water and germinated in 23-cm diameter earthen pots filled with acid-washed sand purified according to Hewitt (1966). The plants were raised in full strength modified Hoagland nutrient solution containing 3 mM KNO_3_, 2 mM Ca(NO_3_)_2_, 1 mM NH_4_H_3_PO_4_, 50 μM KCl, 25 μM H_3_BO_4_, 2 μM MnCl_2_, 20 μM ZnSO_4_, 0.5 μM CuSO_4_, 0.5 μM (NH_4_)_6_Mo_7_O_24_, and 20 μM Na_2_Fe-EDTA, supplied on alternate days. After germination, three healthy plants of nearly equal size in each pot were maintained. The pots were kept in the naturally illuminated green house of the Botany Department, Aligarh Muslim University, Aligarh (India) [average day/night temperatures, 20 ± 3 and 12 ± 2°C, respectively; relative humidity, 60 ± 5%; photosynthetically active radiation (PAR), 750 ± 25 μmol m^-2^ s^-1^; critical photoperiod, 10–12 h]. Experiments were conducted independently to evaluate the effect of 0, 5, 10, and 20 μM MeJA sprayed on plants at 15 days after sowing (DAS) in presence or absence of 50 μM Cd on content of H_2_O_2_ and GSH, photosynthesis, and plant dry mass. Based on the results of this experiment, 10 μM MeJA was selected for spraying on plants grown with or without 1.0 mM ${\mathrm{SO}}_{4}^{2–}$ and 50 μM Cd to determine the effectiveness of MeJA on S-assimilation and GSH production, and alleviation of Cd stress. The concentration 1.0 mM ${\mathrm{SO}}_{4}^{2–}$ was used to initiate S-assimilation and is considered as optimal concentration for mustard (Asgher et al., 2014). Cd concentration used was based on the findings of our previous results (Asgher et al., 2014; Per et al., 2016). To substantiate the role of MeJA-mediated GSH production through S-assimilation in Cd stress alleviation, a GSH biosynthesis inhibitor, BSO (0.5 mM) was added to Cd, MeJA + Cd and MeJA + Cd + S treatments. The effects of the three treatments were compared with the effect of Cd treated plants alone. In experiments, 50 μM Cd or 1.0 mM ${\mathrm{SO}}_{4}^{2–}$ treatment was given along with nutrient solution at 10 DAS and observations were recorded at 30 DAS. The source of Cd was CdCl_2_. MgSO_4_ was used for obtaining 1.0 mM ${\mathrm{SO}}_{4}^{2–}$ and Mg^2+^ was maintained in all treatments including control by the addition of appropriate MgCl_2_ as described earlier (Nazar et al., 2014). Each pot was supplied with 250 mL nutrient solution. The experiment followed a randomized complete block design and was conducted in four replications.

### Determination of Leaf Cd Concentration

Leaf samples were dried for 48 h at 80°C in an oven and the dried tissue was weighed, ground to fine powder and digested with concentrated HNO_3_/HClO_4_ (3:1, v/v). The content of Cd was determined by atomic absorption spectrophotometer (GBC, 932 plus; GBC Scientific Instruments, Braeside, Australia).

### Determination of H_2_O_2_ Content and Lipid Peroxidation

The content of H_2_O_2_ was determined by adopting the method of Okuda et al. (1991) in leaf tissues (0.5 g) grounded in ice-cold 200 mM HClO_4_. The sample was centrifuged at 1200 × *g* for10 min followed by neutralization of HClO_4_ of the supernatant with 4 M KOH. The insoluble KClO_4_ was eliminated by further centrifugation at 500 × *g* for 3 min. In a final volume of 1.5 mL, the reaction mixture contained 1 mL of the eluate, 400 μL of 12.5 mM 3-(dimethylamino) benzoic acid in 0.375 M phosphate buffer (pH 6.5), 80 μL of 3-methyl-2-benzothiazoline hydrazone, and 20 μL of peroxidase (0.25 unit). The reaction was started by the addition of peroxidase at 25°C and the increase in absorbance was recorded at 590 nm.

Lipid peroxidation in leaves was determined in terms of the content of TBARS as described by Dhindsa et al. (1981). Fresh leaf tissues (0.5 g) were ground in 0.25% 2-thiobarbituric acid (TBA) in 10% trichloroacetic acid (TCA) using mortar and pestle. After heating at 95°C for 30 min, the mixture was rapidly cooled on ice bath and centrifuged at 10,000 × *g* for 10 min. To 1 mL of the supernatant, 4 mL 20% TCA containing 0.5% TBA was added. The absorbance of the supernatant was read at 532 nm and corrected for non-specific turbidity by subtracting the absorbance of the same at 600 nm. The content of TBARS was calculated using the extinction coefficient (155 mM^-1^ cm^-1^).

### Determination of S-Assimilation (ATP-S Activity, S Content) and GSH Production

The method of Lappartient and Touraine (1996) was adopted for the measurement of ATP-sulfurylase (ATP-S) activity. The activity of ATP-S was assayed *in vitro* in leaves by measuring molybdate-dependent formation of pyrophosphate. Fresh leaf tissues (1.0 g) were ground at 4°C in buffer consisting of 10 mM Na_2_ EDTA, 20 mM Tris-HCl (pH 8.0), 2 mM dithiothreitol (DTT), and 0.01 g mL^-1^ polyvinylpyrrolidone (PVP), using1:4 (w/v) tissue to buffer ratio. The homogenate was centrifuged at 20,000 × *g* for 10 min at 4°C. The supernatant was used for *in vitro* ATP-S assay. The reaction was initiated by adding 0.1 mL of the extract to 0.5 mL of the reaction mixture, which contained 7 mM MgCl_2_, 5 mM Na_2_MoO_4_, 2 mM Na_2_ ATP, and 0.032 units mL^-1^ of sulfate-free inorganic pyrophosphate in 80 mM Tris-HCl buffer (pH 8.0). Another aliquot from the same extract was added to the reaction mixture but without Na_2_MoO_4_. Incubations were carried out at 37°C for 15 min, after which phosphate was determined using UV-VIS spectrophotometer (LT-2700, UV/VIS Analytical Technologies Limited, Labtronics, India).

For the determination of S content, dried leaf material (0.1 g) was digested in a mixture of concentrated HNO_3_ and 60% strength HClO_4_ (85:15, v/v) and total S in plant samples was determined using turbidimetric method (Chesnin and Yien, 1950). Turbidity in 5 mL aliquot was developed by adding 2.5 mL gum acacia (0.25%) solution, 1.0 g BaCl_2_ sieved through 40–60 mm mesh and the volume was made to 25 mL with de-ionized water. The contents of 25 mL volumetric flask were thoroughly shaken till BaCl_2_ completely dissolved. The values were recorded at 415 nm within 10 min after the turbidity development. A blank was run simultaneously after each set of determination.

Glutathione content was determined following the method of Anderson (1985). Fresh leaves (0.5 g) were homogenized in 2.0 mL of 5% sulphosalicylic acid under cold conditions. The homogenate was centrifuged at 10,000 × *g* for 10 min. To 0.5 mL of supernatant, 0.6 mL of phosphate buffer (100 mM, pH 7.0) and 40 μL of 5′5′-dithiobis-2-nitrobenzoic acid (DTNB) were added. After 2 min the absorbance was read at 412 nm. The details of the procedure are given in our earlier work (Fatma et al., 2014).

### Assay of Antioxidant Enzymes

The activity of SOD was determined using the method of Giannopolitis and Ries (1977) and Beyer and Fridovich (1987), while activity of APX and GR was assayed by the Foyer and Halliwell (1976) and Nakano and Asada (1981), respectively, with slight modifications. Fresh leaf tissues (0.2 g) were homogenized in chilled mortar and pestle with an extraction buffer containing 0.05% (v/v) Triton X-100 and 1% (w/v) polyvinylpyrrolidone in 100 mM potassium-phosphate buffer (pH 7.0). At 4°C, the homogenate was centrifuged at 15,000 × *g* for 20 min. The supernatant obtained after centrifugation was used for the assay of SOD and GR. For the assay of APX extraction buffer was supplemented with 2 mM AsA. The details of procedure have been described earlier in Fatma et al. (2014).

### Measurement of Photosynthetic and Growth Parameters

Gas exchange parameters (net photosynthesis, stomatal conductance, and intercellular CO_2_ concentration) were measured in fully expanded uppermost leaves of plants using infrared gas analyzer (CID-340, Photosynthesis System, Bio-Science, USA). The measurements were done on a sunny day at light saturating intensity; PAR; 780 μmol m^-2^ s^-1^ and atmospheric CO_2_ concentrations; 370 ± 5 μmol mol^-1^.

The activity of ribulose 1,5-bisphosphate carboxylase/oxygenase (Rubisco; EC 4.1.1.39) was determined adopting the method of Usuda (1985) by monitoring NADH oxidation at 30°C at 340 nm. For enzyme extraction, leaf tissue (1.0 g) was homogenized using chilled mortar and pestle with ice-cold extraction buffer containing 0.25 M Tris-HCl (pH 7.8), 0.05 M MgCl_2_, 0.0025 M EDTA and 37.5 mg DTT. This homogenate was centrifuged at 4°C at 10,000 × *g* for 10 min. The resulting supernatant was used to assay the enzyme. The reaction mixture (3 mL) contained 100 mM Tris-HCl (pH 8.0), 40 mM NaHCO_3_, 10 mM MgCl_2_, 0.2 mM NADH, 4 mM ATP, 5 mM DTT, 1U of glyceraldehyde 3-phosphodehydrogenase, 1 U of 3-phosphoglycerate kinase, and 0.2 mM ribulose 1,5-bisphosphate (RuBP).

Chlorophyll content was measured in the intact leaves with the help of SPAD chlorophyll meter (502 DL PLUS, Spectrum Technologies, USA). Chlorophyll *a* fluorescence was measured using Junior-PAM chlorophyll fluorometer (Heinz Walz, Germany). Prior to the measurements of fluorescence intensity, leaf sections were dark-adapted for at least 30 min in order to relax the reaction center. Minimal fluorescence (*F*_o_) and maximum fluorescence (*F*_m_) were measured in dark-adapted leaves with a low measuring beam at a light intensity of 125 μmol m^-2^ s^-1^, whereas under light-adapted condition, minimal fluorescence (*F*_o′_) and maximum fluorescence (*F*_m′_) were measured in the same leaves with a saturating light intensity (780 μmol m^-2^ s^-1^) together with steady-state fluorescence (*F*_s_). The variable fluorescence (*F*_v_ and *F*_v′_) was calculated using the values of *F*_m_-*F*_o_ and *F*_m′_-*F*_o′_, and actual PSII efficiency (ΦPSII) was determined as *F*_m′_-*F*_s_/*F*_m′_, maximal efficiency of PSII by using *F*_v_/*F*_m_ and intrinsic efficiency of PSII by using *F*_v′_/*F*_m′_. Using fluorescence parameters determined in both the light- and dark-adapted states, the photochemical quenching (qP) and non-photochemical quenching (NPQ) parameters were calculated. qP was calculated as (*F*_m′_-*F*_s_)/*F*_v′_ and NPQ as (*F*_m_-*F*_m′_)/*F*_m′_ (Maxwell and Johnson, 2000). Electron transport rate (ETR) was calculated by the following formula: ΦPSII × photosynthetic photon flux density × 0.5 × 0.84 as suggested by Krall and Edwards (1992).

Leaf tissues for chloroplast ultrastructure were prepared for transmission electron microscopy (TEM) by adopting the method of Sandalio et al. (2001) with slight modifications. Leaf samples were cut with razor blade into 1 mm^2^ segments and fixed in 2.5% glutaraldehyde solution in 50 mM phosphate buffer (pH 6.8) for 2.5 h at room temperature. Leaf tissue was then post-fixed for 30 min in 1% osmium tetroxide in 50 mM sodium cacodylate buffer (pH 7.2) and dehydrated in ethanol graded series (30–100%, v/v). After dehydration in a graded series of ethanol, replaced to propylene oxide, and then the tissue was embedded in Spurr resin. Ultrathin sections were taken by using Leica EM UC6 ultramicrotome. Sections were stained with uranyl acetate and lead citrate and examined using a transmission electron microscope (JEOL 2100F, JAPAN) accelerating voltage at 120 kV and magnification at 6000× and 1200×. The chloroplast ultrastructure (thylakoid membranes) was observed from TEM images.

For measuring growth characteristics, plants were uprooted, washed and dried on blotting paper, and were kept in an oven at 80°C till constant weight. The samples were weighed after drying to obtain dry mass. Leaf area was measured using a leaf area meter (LA211, Systronics, New Delhi, India).

### Statistical Analysis

Data were analyzed statistically using analysis of variance (ANOVA) by SPSS 17.0 for Windows, and presented as treatment mean ± SE (*n* = 4). Least significant difference (LSD) was calculated for the significant data at *P* < 0.05 and *P* < 0.01. Bars showing the same letter are not significantly different by LSD test at *P* < 0.05 or *P* < 0.01.

## Results

### MeJA Protects Plants from Cd-Induced Damages

In the absence of Cd, 5 and 10 μM MeJA did not influence content of H_2_O_2_, net photosynthesis and plant dry mass, but 20 μM MeJA significantly increased H_2_O_2_ content and decreased net photosynthesis and plant dry mass as compared to the control. The GSH content remained unchanged with MeJA concentrations as compared to the control (**Table 1**). In the presence of Cd, spraying of 10 μM MeJA was more effective than 5 μM MeJA in reducing H_2_O_2_ content (-31.7%) and increasing GSH content (+52.6%) as compared to the Cd treated plants (**Table 1**).

**Table 1:** Content of H_2_O_2_, net photosynthesis, GSH content, and plant dry mass of mustard (*Brassica juncea* L. cv. Ro Agro 4001) at 30 days after sowing (DAS).

Parameters	Control	5 μM MeJA	10 μM MeJA	20 μM MeJA	50 μM Cd	Cd+5 μM MeJA	Cd+10 μM MeJA	Cd+20 μM MeJA
H_2_O_2_ content (nmol g^-1^ FW)	51.5 @ 3.3ˆdD	49.3 @ 3.3ˆdD	48.6 @ 2.9ˆdD	78.8 @ 3.1ˆcC	108.9 @ 5.3ˆbB	69.6 @ 4.0ˆcC	43.3 @ 4.7ˆdD	120.3 @ 5.6ˆaA
Net photosynthesis (μmol CO_2_ m^-2^ s^-1^)	18.2 @ 1.4ˆbcBC	19.4 @ 1.5ˆb	20.3 @ 0.91ˆabAB	12.7 @ 0.98ˆdeDE	11.1 @ 0.63ˆefEF	15.3 @ 0.67ˆcdCD	22.8 @ 0.89ˆaAAB	8.4 @ 0.36ˆfF
GSH content (nmol g^-1^ FW)	308 @ 13.4ˆdD	312 @ 13.2ˆdD	319 @ 14.6ˆdD	325 @ 14.1ˆdD	363 @ 15.1ˆcC	395 @ 16.3ˆbB	427 @ 17.4ˆaA	373 @ 12.8ˆbcBC
Plant dry mass (g plant^-1^)	2.5 @ 0.21ˆaA	2.52 @ 0.14ˆaA	2.53 @ 0.16ˆaA	2.16 @ 0.06ˆbAB	1.7 @ 0.18ˆbcBC	2.3 @ 0.15ˆaAB	2.7 @ 0.22ˆaA	1.52 @ 0.10ˆdD

*Plants were treated with 0 or 50 μM Cd or 5, 10, and 20 μM MeJA alone or in combination with 50 μM Cd. GSH, reduced glutathione; FW, fresh weight.*

*Data are presented as treatments mean ± SE (*n* = 4). Data followed by same letter are not significantly different by LSD test at *P* < 0.05 and *P* < 0.01. Capital letters indicate significance at *P* < 0.01 and small letters at *P* < 0.05.*

Net photosynthesis and plant dry mass were favorably influenced by MeJA treatment under Cd stress. Application of 10 μM MeJA increased net photosynthesis and plant dry mass by about two-times as compared to the Cd treated plants (**Table 1**).

### MeJA Improves Tolerance to Oxidative Damage Caused by Cd Treatment

**FIGURE 1:**
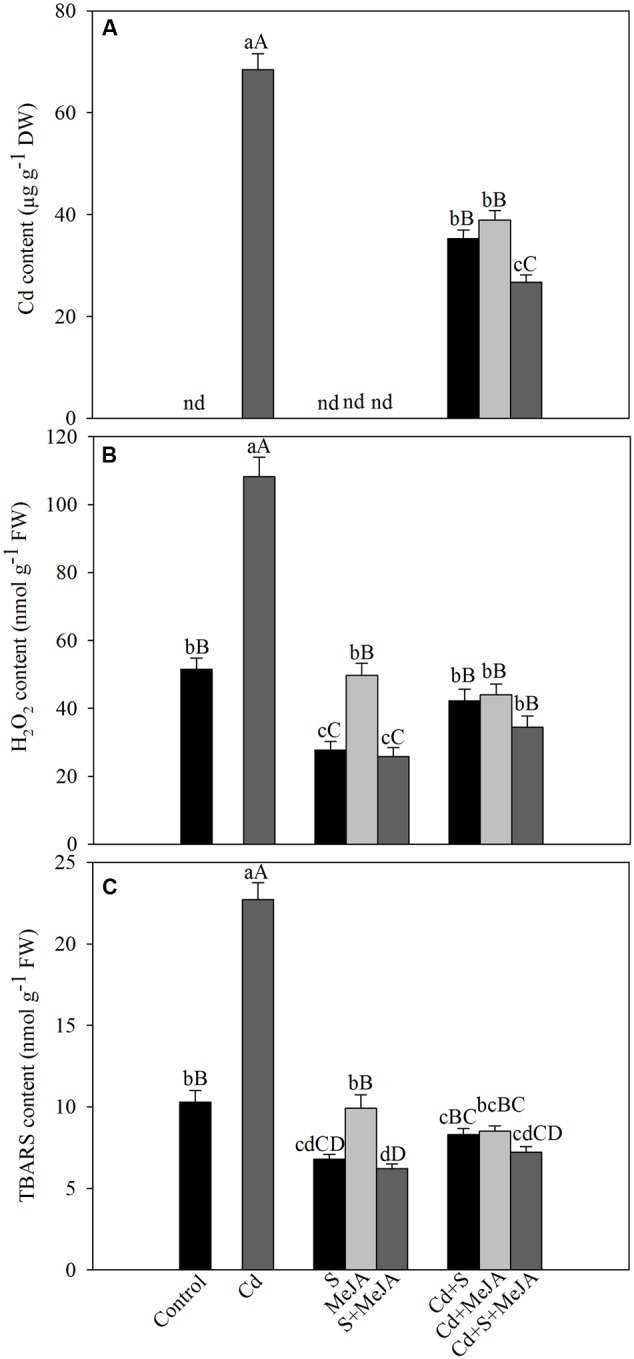
**Content of Cd (A)**, H_2_O_2_ **(B)**, and TBARS **(C)** in mustard (*Brassica juncea* L.) leaves at 30 days after sowing (DAS) in leaves originated from plants treated with/without 50 μM Cd, 1.0 mM ${\mathrm{SO}}_{4}^{2–}$ (S) or 10 μM MeJA individually or in combinations. Data are presented as treatments mean ± SE (*n* = 4). Data followed by same letter are not significantly different by least significant difference (LSD) test at *P* < 0.05 and *P* < 0.01. Capital letters indicate significance at *P* < 0.01 and small letters at *P* < 0.05. nd, not determined; Cd, cadmium; MeJA, methyl jasmonate; TBARS, thiobarbituric acid reactive substances.

To determine the role of MeJA in oxidative stress tolerance, we compared the effect of MeJA on oxidative damage induced by Cd. Application of either MeJA or S reduced Cd content about equally by 93% as compared to the Cd treated plants. The combined treatment of MeJA plus S further reduced the Cd content by 156% in comparison with the Cd treated plants (**Figure 1**).

Plants treated with MeJA or S in the absence of Cd showed that S only could reduce H_2_O_2_ and TBARS contents and the effect of MeJA did not differ with that of control plants. Plants treated with Cd showed an increase of about more than two-times in TBARS content as compared to the control plants. However, treatment with MeJA or S reduced the content of H_2_O_2_ and TBARS in Cd stressed plants almost equally by about 60%, and the combined treatment MeJA + S reduced oxidative stress in Cd stressed plants more prominently, by diminishing H_2_O_2_ and TBARS content by about 70% (**Figure 1**).

**FIGURE 2:**
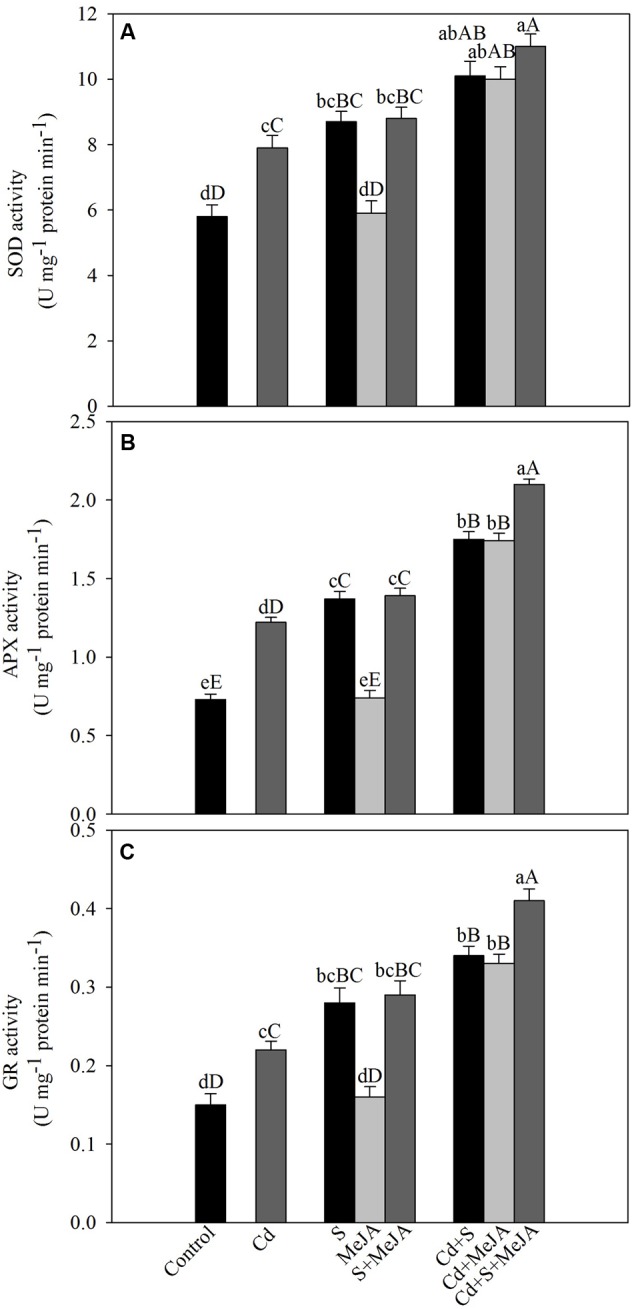
**Activity of SOD (A)**, APX **(B)**, and GR **(C)** in mustard (*B. juncea* L.) leaves treated with 10 μM MeJA and/or grown with 1.0 mM ${\mathrm{SO}}_{4}^{2–}$ (S) in presence or absence of 50 μM Cd at 30 DAS. Data are presented as treatments mean ± SE (*n =* 4). Data followed by same letter are not significantly different by LSD test at *P* < 0.05 and *P* < 0.01. Capital letters indicate significance at *P* < 0.01 and small letters at *P* < 0.05. APX, ascorbate peroxidase; Cd, cadmium; GR, glutathione reductase; MeJA, methyl jasmonate; SOD, superoxide dismutase.

To assess the involvement of antioxidant enzymes in Cd tolerance, activity of SOD, APX, and GR was evaluated under Cd stress. Cd increased the activity of SOD, APX, and GR by 36%, 67%, and 47%, respectively as compared to the control. Application of S in absence of Cd increased activity of antioxidant enzymes, but MeJA application showed no significant changes in the activity of antioxidant enzymes as compared to the control. Plants receiving MeJA or S in the presence of Cd exhibited equal increase in the activity of SOD by about 74%, APX by 140% and GR by 127% as compared to the control. More prominent increase in the activity was found when S was given with MeJA in presence of Cd increasing activity of SOD by 90%, APX by 188% and GR by 173% in comparison with the control (**Figure 2**).

### MeJA Improves S-Assimilation Capacity

**FIGURE 3:**
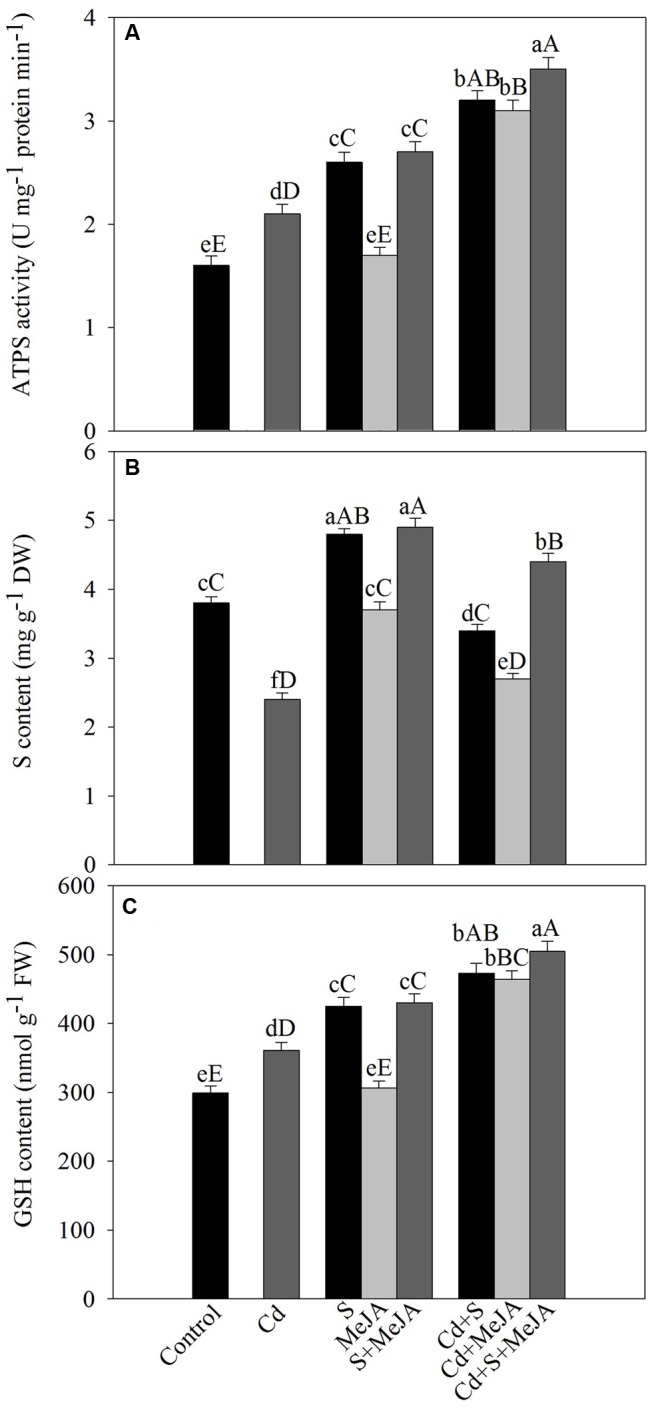
**Activity of ATP-S (A)**, content of S **(B)**, and GSH **(C)** in mustard (*B. juncea* L.) leaves treated with 10 μM methyl jasmonate (MeJA) and/or grown with 1.0 mM ${\mathrm{SO}}_{4}^{2–}$ (S) in presence or absence of 50 μM Cd at 30 DAS. Data are presented as treatments mean ± SE (*n* = 4). Data followed by same letter are not significantly different by LSD test at *P* < 0.05 and *P* < 0.01. Capital letters indicate significance at *P* < 0.01 and small letters at *P* < 0.05. ATP-S, ATP-sulfurylase; Cd, cadmium; GSH, reduced glutathione; MeJA, methyl jasmonate; S, sulfur.

Under Cd stress, activity of ATP-S increased by 31% and S content decreased by 36% in comparison with the control. Application of S or MeJA plus S increased ATP-S activity equally by about 62% and S content by 26% in comparison with the control under non stress condition. MeJA alone did not produce any significant change in the activity of ATP-S and S content as compared to the control. Plants receiving S or MeJA in the presence of Cd showed increase in the activity of ATP-S by about 52% as compared to the Cd treated plants. In presence of Cd, MeJA together with S increased ATP-S activity by 67% as compared to the Cd treated plants (**Figure 3**).

Plants grown with Cd showed significant increase in the content of GSH by 21% as compared to the control. Supplementation of S increased GSH by 40% in the absence of Cd, while MeJA alone showed no significant increase in GSH content in comparison with the control. In the presence of Cd, however, MeJA or S effectively increased GSH content equally by about 30% in comparison with the Cd treated plants. The combined application of MeJA and S in the presence of Cd more prominently increased GSH content by 40% in comparison with the Cd treated plants (**Figure 3**).

### Influence of MeJA on Photosynthetic and Growth Parameters

Cadmium stress severely affected gas exchange parameters, chlorophyll content and Rubisco activity as compared to the control. The plants receiving S showed higher photosynthesis as compared to the control in the absence/presence of Cd. In the presence of Cd, plants receiving S or MeJA completely alleviated the Cd stress and enhanced photosynthesis as compared to the control. In the absence of Cd, plants receiving S showed increase in net photosynthesis by 40%, stomatal conductance by 19%, intercellular CO_2_ concentration by 27% and chlorophyll content by 27% and Rubisco activity by 42% in comparison with the control. However, no significant increase as compared to the control was observed with MeJA application in the absence of Cd. In Cd treated plants, application of MeJA or S was effective in enhancing net photosynthesis by 81 and 102%, stomatal conductance by 32 and 37%, intercellular CO_2_ concentration by 36 and 36%, chlorophyll content by 74 and 83%, and Rubisco activity by 84 and 100%, respectively, as compared to the Cd treated plants. The treatment with MeJA and S together showed more pronounced increase in net photosynthesis by 117%, stomatal conductance by 41%, intercellular CO_2_ concentration by 53%, chlorophyll content by 95%, and Rubisco activity by 117% as compared to the Cd treated plants (**Table 2**).

**Table 2:** Net photosynthesis, stomatal conductance, intercellular CO_2_ concentration, chlorophyll content and Rubisco activity of mustard (*B. juncea* L. cv. Ro Agro 4001) treated with 10 μM methyl jasmonate (MeJA) and/or supplemented with S (1.0 mM ${\mathrm{SO}}_{4}^{2–}$) in presence or absence of 50 μM Cd at 30 DAS.

Treatments	Net photosynthesis (μmol CO_2_ m^-2^ s^-1^)	Stomatal conductance (mmol m^-2^ s^-1^)	Intercellular CO_2_ concentration (μmol mol^-1^)	Chlorophyll content (SPAD value)	Rubisco activity (μmol CO_2_ mg^-1^ (protein) s^-1^)
0	17.3 @ 0.98ˆbb	313 @ 8.9ˆbb	215 @ 4.8ˆcD	35.3 @ 1.70ˆcC	44.8 @ 1.50ˆdD
Cd	10.8 @ 0.70ˆcC	252 @ 8.2ˆcC	171 @ 4.2ˆdE	22.1 @ 1.30ˆdD	27.1 @ 1.18ˆeE
S	24.3 @ 1.10ˆaA	371 @ 7.9ˆaA	273 @ 5.2ˆaA	44.7 @ 1.50ˆaA	63.8 @ 1.60ˆaA
MeJA	17.6 @ 1.22ˆbb	305 @ 8.2ˆbb	221 @ 5.4ˆcCD	36.8 @ 1.40ˆcC	45.9 @ 1.53ˆdD
Cd + S	23.0 @ 1.02ˆaA	346 @ 10.9ˆaA	242 @ 4.6ˆbBC	40.5 @ 1.25ˆbcBC	54.4 @ 1.24ˆbBC
MeJA + Cd	22.6 @ 0.87ˆaA	341 @ 10.3ˆaA	238 @ 6.4ˆbC	38.4 @ 1.24ˆbcBC	52.9 @ 1.57ˆcC
MeJA + S	27.8 @ 1.20ˆaA	375 @ 9.4ˆaA	275 @ 6.2ˆaA	44.9 @ 1.50ˆaA	64.3 @ 1.78ˆaA
MeJA + Cd + S	25.4 @ 1.30ˆaA	362 @ 11.2ˆaA	262 @ 5.0ˆaAB	43.2 @ 1.20ˆabAB	58.7 @ 1.47ˆbAB

*Data are presented as treatments mean ± SE (*n* = 4). Data followed by same letter are not significantly different by LSD test at *P* < 0.05 and *P* < 0.01. Capital letters indicate significance at *P* < 0.01 and small letters at *P* < 0.05.*

**FIGURE 4:**
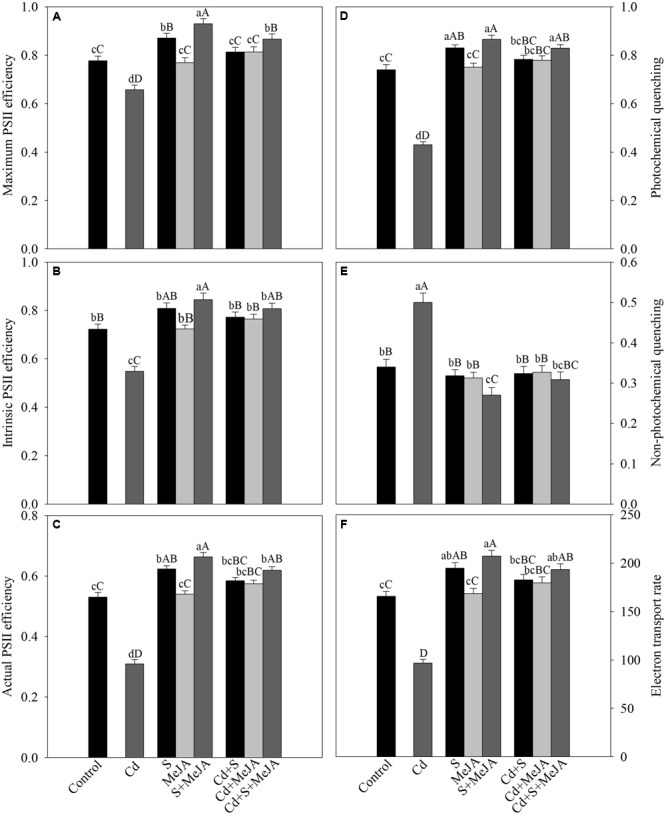
**Maximum PSII efficiency (A)**, intrinsic PSII efficiency **(B)**, actual PSII efficiency **(C)**, photochemical quenching **(D)**, non-photochemical quenching **(E)**, and electron transport rate **(F)** in mustard (*B. juncea* L.) leaves treated with 10 μM MeJA and/or grown with 1.0 mM ${\mathrm{SO}}_{4}^{2–}$ (S) in presence or absence of 50 μM Cd at 30 DAS. Data are presented as treatments mean ± SE (*n* = 4). Data followed by same letter are not significantly different by LSD test at *P* < 0.05 and *P* < 0.01. Capital letters indicate significance at *P* < 0.01 and small letters at *P* < 0.05. Cd, cadmium; MeJA, methyl jasmonate.

The chlorophyll fluorescence parameters varied under different treatments. Plants grown with Cd exhibited reduced maximum PSII efficiency (-13%), intrinsic PSII efficiency (-25%), actual PSII efficiency (-40%), qP (-45%), and ETR (-40%) as compared to the control. However, NPQ increased with Cd stress by 62% as compared to the control. Application of MeJA or S alone improved the above characteristics equally in Cd treated plants in comparison with the Cd treated plants. Application of MeJA and S together more prominently reversed the effect of Cd on chlorophyll fluorescence parameters and resulted in increase in maximum PSII efficiency, intrinsic PSII efficiency, actual PSII efficiency, qP, and ETR by 30%, 51%, 96%, 98%, and 96%, respectively, and decreased NPQ by 46% in comparison with the Cd treated plants (**Figure 4**).

**FIGURE 5:**
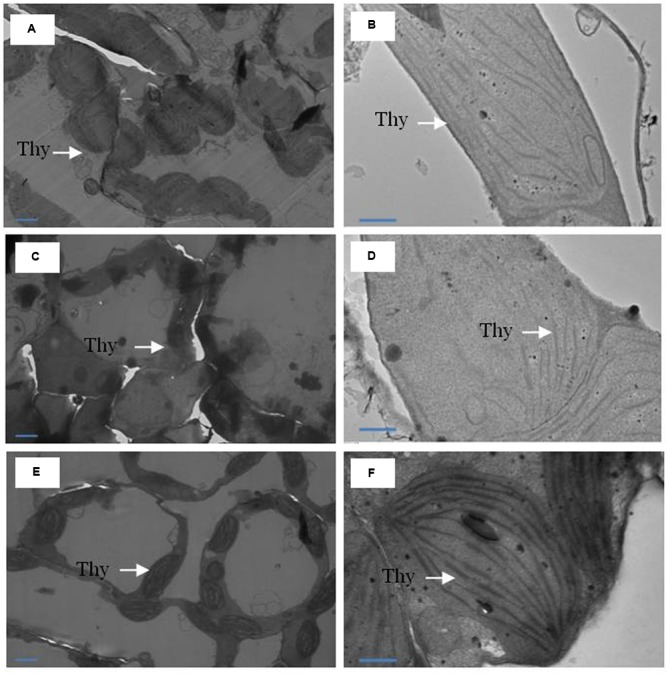
**Ultrastructure of chloroplasts from leaves of mustard (*B. juncea* L.).** Transmission electron microscopy micrographs on the representative chloroplasts from the leaves of mustard performed on the control **(A,B)**; 50 μM Cd **(C,D)** and 1.0 mM ${\mathrm{SO}}_{4}^{2–}$ (S) and 10 μM MeJA with 50 μM Cd **(E,F)** at 30 DAS. Ultrathin sections were prepared, stained with uranyl acetate and lead citrate, and examined by transmission electron microscopy operated at voltage of 120 kV and magnification of 6000× and 1200×. Bar represents 100 nm in the panels **(A,C,E)** and 500 nm in the panels **(B,D,F)**. Thy, thylakoid membranes; Cd, cadmium; MeJA, methyl jasmonate.

The TEM images of ultrastructure of chloroplasts from leaves samples are shown in **Figure 5**. Under normal conditions, chloroplasts were of regular shape with well arranged thylakoid systems. The analysis of TEM of plants grown with Cd showed disturbances in chloroplast ultrastructure. Chloroplast from Cd-treated plants showed disorganized thylakoid systems, however, plants receiving MeJA plus S in the presence of Cd greatly modified the chloroplast ultrastructure and chloroplast showed regular shape with well arranged thylakoid systems containing a markedly increased number of thylakoid stacks (**Figure 5**).

**FIGURE 6:**
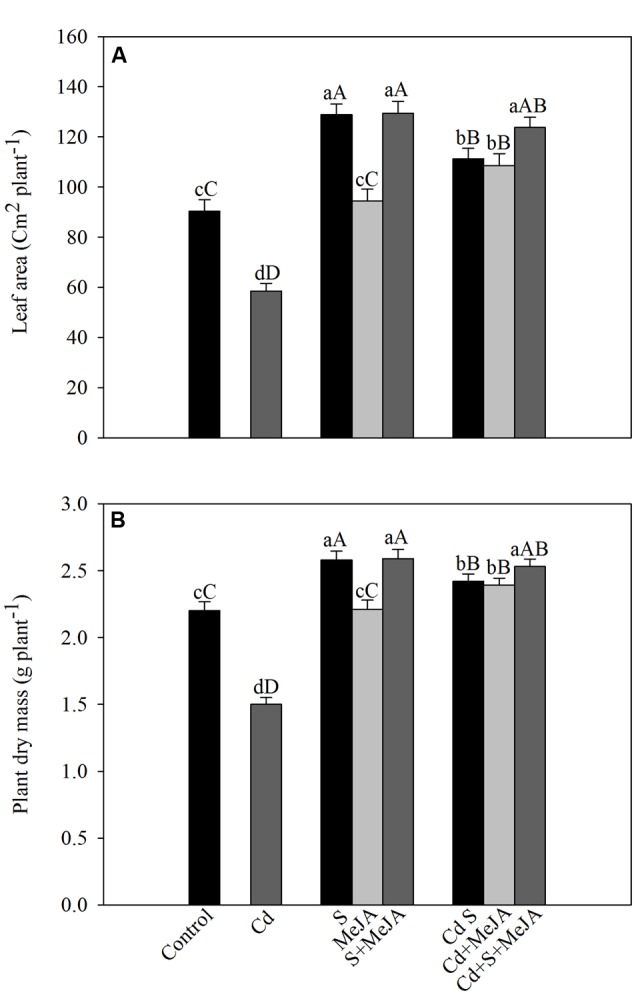
**Leaf area **(A)** and plant dry mass **(B)** of mustard (*B. juncea* L.) leaves treated with 10 μM MeJA and/or grown with 1.0 mM ${\mathrm{SO}}_{4}^{2–}$ (S) in presence or absence of 50 μM Cd at 30 DAS.** Data are presented as treatments mean ± SE (*n* = 4). Data followed by same letter are not significantly different by LSD test at *P* < 0.05 and *P* < 0.01. Capital letters indicate significance at *P* < 0.01 and small letters at *P* < 0.05. Cd, Cadmium; MeJA, methyl jasmonate.

Cadmium stress inhibited the development of leaf area and plant dry mass. Application of S increased leaf area by 43% and plant dry mass by 17% in the absence of Cd in comparison with the control. MeJA alone did not prove effective in increasing leaf area and plant dry mass in the absence of Cd. In the presence of Cd, however, MeJA or S increased leaf area equally by about 90% and plant dry mass by about 60%, respectively, as compared to the Cd treated plants. More prominent increase in leaf area and plant dry mass was noted with combined treatments of MeJA and S (**Figure 6**).

### Effect of BSO on MeJA-Mediated Alleviation of Cd Stress

Application of MeJA lowered the impact of Cd on photosynthetic parameters through increase in production of GSH via S-assimilation. These observations were found when comparison of plants treated with Cd or Cd plus BSO was made with plants receiving BSO in presence of MeJA and Cd or MeJA, Cd and S. Supplementation of BSO to plants treated with MeJA in presence of Cd or MeJA in presence of Cd and S resulted in more negative effects on photosynthetic and growth parameters in comparison with the Cd treated plants. Plants raised under these treatments exhibited enhanced H_2_O_2_ content and lowered GSH content in comparison with the Cd treated plants. However, addition of BSO to Cd treated plants resulted in greatest H_2_O_2_ content and lowest GSH content with highest decrease in photosynthesis and growth as compared to the other treatments. The plants treated with S in presence of MeJA, Cd, and BSO showed lesser decrease in photosynthesis and growth in comparison with the plants treated with MeJA, Cd, and BSO (**Table 3**).

**Table 3:** Content of H_2_O_2_, GSH, net photosynthesis, stomatal conductance, intercellular CO_2_ concentration, chlorophyll content, Rubisco activity, PSII efficiency, leaf area and plant dry mass of mustard (*B. juncea* L. cv. Ro Agro 4001) treated with 50 μM Cd, 50 μM Cd + 0.5 mM BSO or 50 μM Cd + 10 μM MeJA + 0.5 mM BSO or 50 μM Cd + 1.0 mM ${\mathrm{SO}}_{4}^{2–}$ + 10 μM MeJA + 0.5 mM BSO at 30 DAS.

Parameters	Cd	Cd + BSO	MeJA + Cd + BSO	MeJA + Cd + S + BSO
H_2_O_2_ content (nmol g^-1^ FW)	105.4 @ 5.1ˆdD	216.4 @ 3.99ˆaA	180.3 @ 3.95ˆbB	138.2 @ 4.03ˆcC
GSH content (nmol g^-1^ FW)	371 @ 10.2ˆaA	163 @ 7.4ˆdD	194 @ 8.2ˆcC	218 @ 9.4ˆbB
Net photosynthesis (μmol CO_2_ m^-2^ s^-1^)	15.8 @ 0.69ˆaA	6.0 @ 0.12ˆdC	6.6 @ 0.26ˆcC	8.1 @ 0.26ˆbB
Stomatal conductance (mmol m^-2^ s^-1^)	258 @ 8.8ˆaA	172 @ 5.0ˆdC	205 @ 4.8ˆcBC	229 @ 5.2ˆbB
Intercellular CO_2_ concentration (μmol mol^-1^)	181 @ 6.6ˆaA	110 @ 4.1ˆdC	135 @ 4.2ˆcBC	155 @ 4.4ˆbB
Chlorophyll content (SPAD value)	23.2 @ 1.1ˆaA	11.1 @ 0.73ˆdD	12.8 @ 0.69ˆcC	16.5 @ 0.62ˆbB
Rubisco activity (μmol CO_2_ mg^-1^ (protein) s^-1^)	27.9 @ 1.12ˆaA	16.1 @ 0.59ˆdD	18.0 @ 0.49ˆcC	25.2 @ 0.51ˆbB
PSII efficiency (*F*_v_/*F*_m_)	0.659 @ 0.0126ˆaA	0.419 @ 0.0104ˆdD	0.512 @ 0.0122ˆcC	0.609 @ 0.013ˆbB
Leaf area (cm^2^ plant^-1^)	60.3 @ 2.8ˆaA	23.6 @ 2.0ˆdD	33.3 @ 2.3ˆcC	49.4 @ 2.3ˆbB
Plant dry mass (g plant^-1^)	1.53 @ 0.05ˆaA	1.08 @ 0.04ˆdD	1.16 @ 0.04ˆcC	1.27 @ 0.04ˆbB

*BSO, buthionine sulfoximine; FW, fresh weight; GSH, reduced glutathione; MeJA, methyl jasmonate.*

*Data are presented as treatments mean ± SE (*n* = 4). Data followed by same letter are not significantly different by LSD test at *P* < 0.05 and *P* < 0.01. Capital letters indicate significance at *P* < 0.01 and small letters at *P* < 0.05.*

## Discussion

### Effect of MeJA on Cd Stress Tolerance

The lipid peroxidation and H_2_O_2_ generation are indicators of oxidative stress and the general outcome of metal toxicity (Srivastava et al., 2014; Khan et al., 2015). Lipid peroxidation and H_2_O_2_ content significantly increased with the increase of Cd accumulation in Cd treated plants (**Figure 1**). Addition of MeJA plus S, however, maximally decreased contents of H_2_O_2_ and TBARS showing the ability of MeJA in alleviation of Cd-induced oxidative stress. These findings are supported by the previous results of Singh and Shah (2014), Hanaka et al. (2015) and Farooq et al. (2016). Plants under oxidative stress induce synthesis of antioxidant metabolites and activity of antioxidant enzymes for protection against oxidative stress. In this study, the application of MeJA and S in Cd-stressed plant increased the activity of antioxidant enzymes more than their individual application (**Figure 2**) and substantially decreased ROS (**Figures 1B,C**). Application of 5 μM MeJA in *O. sativa* (Singh and Shah, 2014), 0.1–1.0 μM MeJA in *Capsicum frutescens* (Yan et al., 2013), 0.1–10 μM MeJA in *Kandelia obovata* (Chen et al., 2014), and 0.1 μM MeJA in *Solanum nigrum* (Yan et al., 2015) has resulted in increased activity of antioxidant enzymes and Cd tolerance. Furthermore, application 0.1–10 μM MeJA minimized oxidative stress in As exposed *B. napus* plants by enhancing enzymatic activity and gene expression of antioxidant enzymes and also by regulating multiple transcriptional pathways involved in oxidative stress responses (Farooq et al., 2016).

### MeJA Enhances S-Assimilation and GSH Production for Cd Tolerance

Application of MeJA or/and S increased activity of ATP-S and content of S and GSH under Cd stress (**Figure 3**). Studies have shown that thiol metabolism and antioxidant defense system increased in *Hordeum vulgare* plants subjected to metals stress (Astolfi et al., 2012). The results reported here have suggested that ATP-S plays a key role in maintaining GSH pool required for Cd stress tolerance. The potential of plants to survive in Cd contaminated conditions has been found to improve by up-regulation of S-assimilation pathway (Wangeline et al., 2004; Bashir et al., 2013). Under normal conditions, GSH level is maintained in plant cells in a steady state via a low level of transcription, translation, and optimal enzyme activity. However, when plants are subjected to stress, the homeostasis is perturbed and GSH pool is consumed to combat stress. The increased GSH production in the presence of MeJA as observed in the present study in Cd-stressed plant is in close conformity to the results of Singh and Shah (2014) in *O. sativa* under Cd stress and Farooq et al. (2016) in *B. napus* under As stress, who found that antioxidant system was stimulated with MeJA. Plants with improved capacity for GSH synthesis have been found to display higher Cd-tolerance (Schützendübel et al., 2002; Yan et al., 2015). Xiang and Oliver (1998) have reported that JA treatment increased the transcript levels of γ-ECS (GHS1) and GSH2 as well as GR and also several GSH-S-transferase encoding genes (Schenk et al., 2000; Sasaki et al., 2001).

### MeJA Improves Photosynthesis and Growth under Cd Stress

The increase in Rubisco activity with MeJA suggests restoration of photosynthetic apparatus in Cd-stressed plants. The role of MeJA in protecting plants under various abiotic stresses is controversial. However, our results have shown that individual or combined application of MeJA and S favored S-assimilation and GSH synthesis. This cumulatively protected chloroplast ultrastructure. Iqbal et al. (2012) have shown the relationship between S allocation in leaves and Rubisco activity. The decreased chlorophyll content in response to Cd supply was related to disorganization in chlorophyll biosynthesis (Parmar et al., 2013). MeJA at 100 μM or higher concentration repressed germination (Kobayashi et al., 2010) and plant growth (Jubany-Marí et al., 2010), Rubisco genes, chlorophyll *a*/*b*-binding protein, light harvesting complex II (Jung et al., 2007). In contrast, lower MeJA concentration mostly stimulated plant growth, lateral root initiation, dry matter accumulation, photosynthetic pigment levels, and net photosynthetic rate (Tung et al., 1996; Mabood et al., 2006; Piotrowska et al., 2010; Wu et al., 2012). Our study showed that 10 μM MeJA improved chlorophyll content, gas exchange parameters and Rubisco activity under Cd stress. This was associated with the activation of antioxidant enzymes and recovery of photosynthetic efficiency. Keramat et al. (2010) have also reported that MeJA at 0.01 mM was more effective in reducing the damages of Cd stress on shoot dry weight and total chlorophyll content in *G. max*. Recently, it has been shown that S and Se increased photosynthesis via increase in the allocation of N and S to Rubisco in *B. juncea* and *Triticum aestivum* crops (Fatma et al., 2014; Khan et al., 2015).

The drop in chlorophyll fluorescence under HMs stress results from destruction of antenna pigments by the partial block of electron transport from PSII to PSI (Krupa et al., 1993). The application of MeJA together with S prevented the chloroplast structural damage under Cd stress (**Figure 5**). It has been suggested that the lipid-to-chlorophyll ratio is a good estimate for the protein-packing density in thylakoid, and the high lipid-to-chlorophyll ratio reflects a low protein-packing density (Kirchhoff, 2013). In the present study, plants receiving MeJA and S exhibited lower level of lipid peroxidation and showed higher chlorophyll content than control or Cd treated plants. A coordinated impact of MeJA and S on the ultrastructural changes of chloroplasts under Cd stress has not been described earlier.

The increase in growth with MeJA or/and S is attributed to GSH-mediated changes in photosynthesis. The maximum alleviation of Cd stress on growth was observed with the combined treatment of MeJA plus S apparently because of more efficient S-assimilation and GSH production that resulted in maximum protection of cell (**Figure 6**). MeJA treatment has also been reported to reduce the toxic effects of Cd and restored plant growth (Keramat et al., 2009; Kováčik et al., 2011; Yan et al., 2015). Similarly, 0.001 and 0.01 mM MeJA alleviated Cu and Cd ions toxicity on growth in *A. thaliana* (Maksymiec and Krupa, 2002; Yan et al., 2013).

### Involvement of GSH in MeJA-Mediated Alleviation of Cd Stress and Protection of Photosynthesis

There was involvement of MeJA in production of GSH through its effect on S-assimilation and protection of photosynthesis under Cd stress. MeJA enhanced S utilization in GSH synthesis under Cd stress, so that the highest GSH content was observed in Cd + S + MeJA treated plants. The same plants showed also the highest ATP-S activity (**Figure 3**), the lowest Cd content among stressed plants (**Figure 1A**), and the highest antioxidant response (**Figure 2**). Application of BSO to plants treated with Cd reduced production of GSH and inhibited photosynthesis, but GSH production and photosynthesis were higher in plants receiving MeJA or MeJA and S under Cd stress (**Table 3**). This reduction in GSH production aggravated oxidative damage significantly by increasing H_2_O_2_ production and consequently reduced photosynthesis and growth characteristics. In the presence S, MeJA stimulated GSH production via increased S-assimilation under Cd even with the supplementation of BSO. It has been suggested that MeJA either up-regulated the expression of genes involved in biosynthesis of GSH or facilitated the synthesis of GSH (Xiang and Oliver, 1998; Farooq et al., 2016) and enhanced peroxide removal and eventually photosynthetic efficiency under Cd stress. Therefore, MeJA-mediated protection of photosynthesis under Cd stress was correlative with GSH production.

## Conclusion

In conclusion, it may be said that application of MeJA and S prominently mitigated the adverse effects of Cd stress on *B. juncea* through increase in S-assimilation and GSH production and promoted photosynthesis, chlorophyll fluorescence and growth. The application of MeJA induced tolerance in *B. juncea* plants against Cd stress by maintaining production of GSH which played a key role in scavenging ROS. These findings were substantiated with the use of BSO that showed that plants treated with BSO prominently reversed the effects of MeJA with or without S. Further studies are needed to elucidate how MeJA-induced GSH biosynthesis for alleviation of Cd stress.

## Author Contributions

TP designed and conducted the experiment, AM, MF helped in data analysis and presentation, while NK overall supervised the work and corrected the manuscript.

## Conflict of Interest Statement

The authors declare that the research was conducted in the absence of any commercial or financial relationships that could be construed as a potential conflict of interest.
